# An *elegans* Solution for Crossover Formation

**DOI:** 10.1371/journal.pgen.1003658

**Published:** 2013-07-18

**Authors:** Stephanie P. Bellendir, Jeff Sekelsky

**Affiliations:** 1 Curriculum in Genetics and Molecular Biology, University of North Carolina, Chapel Hill, North Carolina, United States of America; 2 Department of Biology, University of North Carolina, Chapel Hill, North Carolina, United States of America; National Cancer Institute, United States of America

*The MUS-81, SLX-1, and XPF-1 structure-selective endonucleases have been implicated in meiotic crossover (CO) formation in a variety of organisms, but their contributions to C. elegans CO formation have been unclear. In this issue of PLOS Genetics, Agostinho et al., Saito et al., and O'Neil et al. demonstrate that MUS-81 and XPF-1 function in two parallel pathways during the formation of meiotic crossovers in C. elegans and provide important insights into the interplay between endonucleases and the Bloom helicase ortholog during crossover formation.*

Meiotic crossovers are important for chromosome segregation. When COs are absent or misplaced, nondisjunction results in aneuploidy, a leading cause of miscarriages and birth defects in humans. Our understanding of the complex process that generates COs has evolved steadily, and the models describing the molecular details have undergone much editing. Key features of the most commonly cited model for meiotic recombination include initiation by a DNA double-strand break (DSB) and a progression of joint molecule (JM) intermediates that link DNA duplexes on homologous chromosomes (reviewed in [1]). In this model, COs are produced by cleavage of a late JM intermediate that has two Holliday junctions (HJs). This cleavage is accomplished by HJ resolvases. A wealth of studies in the yeast *Saccharomyces cerevisiae* have provided extensive genetic and biochemical support for this model. Although initiation by DSBs appears to be universal (reviewed in [2]), it is unknown how similar subsequent steps are in other organisms. This issue of *PLOS Genetics* contains a set of three papers that describe progress toward answering this question in *C. elegans*, particularly with regard to functions of resolvases in generating COs [3]–[5] (Figure 1).

**Figure 1:**
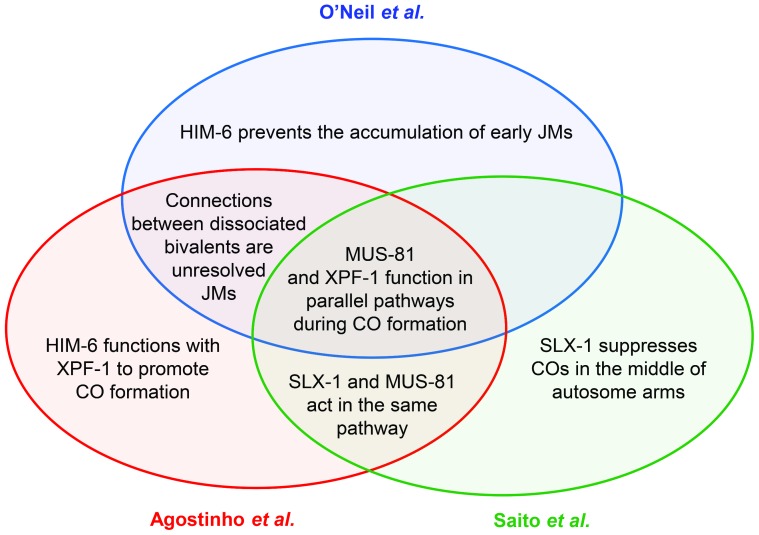
Venn diagram summarizing the main conclusions shared between the three publications discussed in the text.

Numerous searches for *S. cerevisiae* enzymes with *in vitro* resolvase activity eventually identified three structure-selective endonucleases: Mus81–Mms4, Yen1, and Slx1–Slx4 [6]–[8]. Mus-81–Mms4 and Yen1 are important in generating mitotic COs [9]. Elucidating the functions of these enzymes in generating meiotic crossovers has been more challenging, but substantial progress has been made recently [10], [11]. Our current understanding is that there are at least two CO pathways in *S. cerevisiae* meiosis (reviewed in [1]). The major pathway produces interfering COs, which are located farther apart than at random. The resolvase that generates these COs is thought to include Exo1 and the MutLγ complex, proteins that are also involved in mismatch repair [11]. Mus81–Mms4, Yen1, and Slx1–Slx4 do function in CO formation, but through a secondary pathway that yields non-interfering COs. Among single mutants, however, only *mus81* and *mms4* have reduced COs, and even loss of all three confers a relatively modest defect in JM resolution and CO formation, due to redundant and compensatory activities [10], [11].

In the nematode *C. elegans*, previous work found that SLX-1 and the Slx4 ortholog HIM-18 are required for some meiotic COs [12]. These studies also found a role for the nucleotide excision repair endonuclease XPF-1, which was examined because of the prominent role for its *Drosophila* ortholog, MEI-9, in generating meiotic COs [13]. To shed additional light on the relationships between the proteins responsible for CO formation in *C. elegans*, Agostinho *et al.*, O'Neil *et al.*, and Saito *et al.* [3]–[5] quantified numerous meiotic phenotypes in single, double, triple, and even quadruple mutants. These studies led to the discovery of two parallel, partially redundant pathways—one dependent on MUS-81 and the other on XPF-1. Agostinho *et al.* and Saito *et al.* additionally demonstrate that SLX-1 functions with MUS-81 and that HIM-18 functions in both pathways, and Agostinho *et al.* provide evidence that HIM-6, the ortholog of the Bloom syndrome helicase, collaborates with XPF-1. Saito *et al.* also extend a previous finding that SLX-1 actually prevents COs in the centers of the chromosomes [12].

What happens in the absence of both pathways? All three groups report multiple chromosome abnormalities. Most strikingly, connections between homologs persist into diakinesis, where they appear as fine DAPI-stained bridges. The researchers hypothesize that these bridges result from unresolved JMs. To test this hypothesis, Agostinho *et al.* and O'Neil *et al.* removed SPO-11, the enzyme that generates DSBs that initiate meiotic recombination. This eliminated chromatin bridges. In addition, O'Neil and colleagues elegantly show that the chromatin bridges in *mus-81;xpf-1* mutants are efficiently resolved by germline injection of human GEN1, which cleaves HJs *in vitro* [7], providing further support for the hypothesis that the bridges are due to unresolved HJs.

These studies further highlight the substantial complexity among resolvase functions in meiosis. Although *S. cerevisiae* resolvase functions are similarly complex, the details are different in many ways. First, in *S. cerevisiae*, Mus81 generates only non-interfering COs, but *C. elegans* MUS-81 seems to be involved in making interfering COs. Second, *S. cerevisiae* Rad1, the ortholog of XPF-1, has no apparent meiotic function [11]. Third, the JM-resolving activity of *S. cerevisiae* Slx1 and Slx4 is evident only in certain mutant backgrounds [10], [11]. Fourth, in *S. cerevisiae*, there is no evidence that a resolvase can have an anti-CO role, as is suggested by Saito *et al.* for SLX-1 at the centers of chromosomes [4].

Additional potential similarities arise from studies of *him-6*, which encodes the ortholog of human Bloom syndrome helicase (BLM) and *S. cerevisiae* Sgs1. Sgs1 disassembles early meiotic JMs, both to generate noncrossovers and to prevent formation of aberrant multi-chromatid JMs [10], [11]. O'Neil *et al.* report that *him-6* mutants have defects in processing early intermediates, leading to chromosome fragmentation [3]. Agostinho *et al.* propose that HIM-6 also has a late function in generating COs in conjunction with XPF-1 [5]. This is an exciting idea, especially given the recent discovery of pro-CO roles for Sgs1 and *Drosophila* BLM [10], [11], [14], though the authors caution that a full understanding of this function is complicated by the earlier function for HIM-6.

Although the three groups began with similar goals, the different approaches taken in the three papers complement one another to provide numerous important insights into resolvase functions in *C. elegans* meiosis (Figure 1). The results raise several important questions for future studies. First, what is the source of the COs that occur when both the MUS-81 and XPF-1 pathways are missing? Saito *et al.* and Agostinho *et al.* find that COs are reduced by only a little more than one third in *mus-81*;*xpf-1*. A simple interpretation is that neither of these enzymes identifies the major meiotic resolvase, but the truth is likely to be more complex. The assays used to measure COs necessarily select for a distinct subset of the progeny: those that had enough COs to ensure proper chromosome segregation. It is possible that the real decrease is more severe, and that MUS-81 and XPF-1 do define the major resolvases. Either way, there must be at least one additional resolvase that hasn't been identified. The authors of these papers discuss several candidates that might be tested.

Another important question concerns the exact nature of the pre-crossover JM. The authors suggest that these resolvases are acting on HJs, and resolution of chromatin bridges by GEN1 supports this suggestion. Agostinho *et al.* propose intriguing ideas for how the sets of enzymes (MUS-81, SLX-1, and HIM-18; and XPF-1, HIM-18, and HIM-6) might work together to resolve HJs. However, it is possible that the proteins act on a different structure, such as nicked HJs (*e.g.*, see reference [8]). Either way, the groundwork laid in this set of papers will facilitate future experiments aimed at answering these questions and many others.

## References

[1] Kohl KP, Sekelsky J (2013) Meiotic and mitotic recombination in meiosis. Genetics 194: 327–334. 23733849 10.1534/genetics.113.150581 PMC3664844

[2] Keeney S (2008) Spo11 and the formation of DNA double-strand breaks in meiosis. Genome Dyn Stab 2: 81–123. 21927624 10.1007/7050_2007_026 PMC3172816

[3] O'Neil NJ, Martin JS, Youds JL, Ward JD, Petalcorin MIR, et al (2013) Joint molecule resolution requires the redundant activities of MUS-81 and XPF-1 during *Caenorhabditis elegans* meiosis. PLoS Genet 9: e1003582 doi:10.1371/journal.pgen.1003582 23874209 10.1371/journal.pgen.1003582 PMC3715453

[4] Saito TT, Lui DY, Kim H-M, Meyer K, Colaiácovo MP (2013) Interplay between structure-specific endonucleases for crossover control during *Caenorhabditis elegans* meiosis. PLoS Genet 9: e1003586 doi:10.1371/journal.pgen.1003586 23874210 10.1371/journal.pgen.1003586 PMC3715419

[5] Agostinho A, Meier B, Sonneville R, Jagut M, Woglar A, et al (2013) Combinatorial regulation of meiotic Holliday junction resolution in *C. elegans* by HIM-6 (BLM) helicase, SLX-4, and the SLX-1, MUS-81 and XPF-1 nucleases. PLoS Genet 9: e1003591 doi:10.1371/journal.pgen.1003591 23901331 10.1371/journal.pgen.1003591 PMC3715425

[6] Kaliraman V, Mullen JR, Fricke WM, Bastin-Shanower SA, Brill SJ (2001) Functional overlap between Sgs1-Top3 and the Mms4-Mus81 endonuclease. Genes Dev 15: 2730–2740. 11641278 10.1101/gad.932201 PMC312806

[7] Ip SC, Rass U, Blanco MG, Flynn HR, Skehel JM, et al (2008) Identification of Holliday junction resolvases from humans and yeast. Nature 456: 357–361. 19020614 10.1038/nature07470

[8] Schwartz EK, Heyer WD (2011) Processing of joint molecule intermediates by structure-selective endonucleases during homologous recombination in eukaryotes. Chromosoma 120: 109–127. 21369956 10.1007/s00412-010-0304-7 PMC3057012

[9] Ho CK, Mazon G, Lam AF, Symington LS (2010) Mus81 and Yen1 promote reciprocal exchange during mitotic recombination to maintain genome integrity in budding yeast. Mol Cell 40: 988–1000. 21172663 10.1016/j.molcel.2010.11.016 PMC3021384

[10] De Muyt A, Jessop L, Kolar E, Sourirajan A, Chen J, et al (2012) BLM helicase ortholog Sgs1 is a central regulator of meiotic recombination intermediate metabolism. Mol Cell 46: 43–53. 22500736 10.1016/j.molcel.2012.02.020 PMC3328772

[11] Zakharyevich K, Tang S, Ma Y, Hunter N (2012) Delineation of joint molecule resolution pathways in meiosis identifies a crossover-specific resolvase. Cell 149: 334–347. 22500800 10.1016/j.cell.2012.03.023 PMC3377385

[12] Saito TT, Youds JL, Boulton SJ, Colaiácovo MP (2009) *Caenorhabditis elegans* HIM-18/SLX-4 interacts with SLX-1 and XPF-1 and maintains genomic integrity in the germline by processing recombination intermediates. PLoS Genet 5: e1000735 doi:10.1371/journal.pgen.1000735 19936019 10.1371/journal.pgen.1000735 PMC2770170

[13] Sekelsky J, McKim KS, Chin GM, Hawley RS (1995) The Drosophila meiotic recombination gene *mei-9* encodes a homologue of the yeast excision repair protein Rad1. Genetics 141: 619–627. 8647398 10.1093/genetics/141.2.619 PMC1206761

[14] Kohl KP, Jones CD, Sekelsky J (2012) Evolution of an MCM complex in flies that promotes meiotic crossovers by blocking BLM helicase. Science 338: 1363–1365. 23224558 10.1126/science.1228190 PMC3599781

